# Case report: Evaluation of head trauma in a tawny owl (*Strix aluco*) with advanced imaging diagnostic, FVEP and BAER test

**DOI:** 10.3389/fvets.2024.1439432

**Published:** 2024-08-22

**Authors:** Alessandro Vetere, Nicola Della Camera, Ciro Cococcetta, Carlo Paoletti, Maurizio Dondi, Fabio Biaggi, Francesco Di Ianni

**Affiliations:** ^1^Department of Veterinary Science, Università Degli Studi di Parma, Parma, Italy; ^2^Clinica Veterinaria Pedrani, Zugliano, Italy; ^3^Department of Exotic Animals, Centre Hospitalier Vétérinaire Saint-Martin, Allonzier-la-Caille, France; ^4^Department of Exotic Animals, Centre Hospitalier Vétérinaire ADVETIA, Vélizy-Villacoublay, France

**Keywords:** raptors, birds of prey, head trauma, BAER, tawny owl, FVEP, advanced imaging

## Abstract

An adult pet tawny owl (*Strix aluco*) presented to a veterinary hospital at Parma University with a history of head trauma. After a critical care protocol including thermal, oxygen and fluid support aimed at stabilizing the patient, a neurological examination was performed. During neurological evaluation, marked lethargy and an inability to rise from a recumbent position was noted. Anisocoria was also present, with a mydriatic left pupil exhibiting no pupillary light response (PLR) even on direct illumination of both eyes. On ocular fundus examination, retinal hemorrhage and retinal detachment were observed. Based on these clinical findings, a complete work-up was performed, including hematological exams and total body X-ray studies followed by a computed tomography (CT) scan. Additional examinations, such as brainstem auditory evoked response (BAER) measurement and flash visual evoked potential (FVEP) recording, were performed. FVEP measurements performed on the left eye exhibited no peaks in either series of stimulations, indicating an altered functional integration within the visual pathway. A CT scan revealed a large hypoattenuating lesion within the right cerebral hemisphere, suspected to be intraparenchymal edema. The BAER test demonstrated an altered trace consistent with brainstem involvement and left hypoacusis due to cranial nerve VIII deficiency. Head trauma can result in significant neurological impairments in birds, impacting their behavior, mobility, and cognitive abilities. FVEP recordings, BAER tests and CT scans may be useful diagnostic tools in clinical practice. Understanding the causes and neurologic presentation of avian traumas is essential for effective prevention, diagnosis and treatment of affected birds.

## 1 Introduction

In wild birds, common types of trauma include cuts, abrasions, fractures, punctures, sprains, dislocations, concussions, and internal organ damage (1). The causes of bird trauma are multifaceted and can be broadly categorized into natural and anthropogenic factors (2). Natural causes include predator attacks, territorial disputes, adverse weather conditions, and collisions with trees in flight. Anthropogenic causes encompass a range of human activities, such as electrocution from power lines, vehicle collisions, entanglement in man-made structures such as nets and fences or collisions against glass or windows (1–3). Traumas can result in neurological manifestations in birds, leading to a range of behavioral, sensory, motor, and cognitive impairments. Common neurologic presentations in birds include altered responsiveness, abnormal posture or movement, impaired flight or balance, loss of coordination, abnormal reflexes, and sensory deficits (2–4). Accurate diagnosis through thorough neurologic examinations, advanced imaging techniques, and laboratory tests can guide appropriate treatment strategies, including supportive care, pain management, surgical interventions, and rehabilitation (5). Recently, Foss et al. (6) established a Modified Glasgow Coma Scale for avian species to serve as a prognostic indicator in raptors with head trauma. This proposed scale demonstrated good to excellent inter-rater agreement among evaluators from diverse backgrounds in assessing raptors with head trauma. Furthermore, it has been correlated with the probability of survival within the first 48 h after presentation to rehabilitation facilities for raptors suffering from head trauma.

Computed tomography (CT) scanning has emerged as a powerful imaging modality, providing detailed cross-sectional images of avian patients (7, 8). CT scanning involves the use of an x-ray tube revolving around the subject while opposed detectors quantify how much of the x-ray beam is attenuated as it passes through the body. These attenuation values are processed by a computer algorithm to construct detailed cross-sectional images, or slices, of the bird’s body. CT scans play a crucial role in the assessment and management of head traumas in birds, as they provide detailed imaging of cranial structures, facilitating the identification and characterization of traumatic lesions (4, 8). By visualizing bone, soft tissue, and the brain, CT scans aid in detecting fractures, skull deformities, hemorrhages, contusions, foreign bodies, and other traumatic abnormalities (4, 9, 10). These images enable clinicians to assess the extent and localization of the injuries, guiding appropriate treatment decisions and surgical interventions (4, 7). Magnetic resonance imaging (MRI) is ideally suited for soft tissue assessment because of its excellent soft tissue contrast and its ability to image directly in any plane. However, MRI comes with higher costs, is less widely accessible, and typically necessitates longer periods of anesthesia (8). Moreover, the very small size of the brain in most birds results in poor anatomic detail (8, 10, 11).

Brainstem auditory evoked response (BAER) tests are non-invasive and evaluate components of the external ear canal; middle and inner ear cavities; cranial nerves VIII (BAER and the afferent limb of the acoustic reflex), V and VII (both involved in the afferent branches of the acoustic reflex); and.

selected areas of the brainstem and cerebral cortex (12, 13). In fact, the results obtained in conscious and unconscious patients are similar; therefore, this test can be useful to better specify the degree of involvement of brainstem structures during sensory obtundation (13). In practice, BAER measurement requires three electrodes (recording, ground, and reference), an amplifier, and a stimulator (12). The response to the stimuli is recorded as a series of peaks (waves I–V) at approximately 1 ms intervals. The origin of peak I (eighth pair of cranial nerves) is usually easily recognized; peak II arises in the cochlear nucleus, peak III in the inferior olive complex and peaks IV and V in the inferior colliculus (11). Several studies have been conducted on the use of the BAER test in healthy birds, highlighting different responses with respect to the perception of sound waves in nocturnal and diurnal birds, birds of prey and those born in captivity (10–16).

Flash visual evoked potentials (FVEPs) are non-invasive electrodiagnostic tests used to diagnose neurological disorders (17, 18). FVEPs have clinical applications in ophthalmological retinal pathology and neurological pathology related to the optic nerve and/or brain. FVEPs allow the detection of altered functional integration within the visual pathway originating from the retina to the cortical areas through the optic nerve as a result of light stimulation. The triggering of these neuro-anatomic pathways is represented on the recorded waveforms as a series of distinct peaks (positive or negative) according to the variation in the electric field over time. In birds, the use of FVEPs was demonstrated in pigeons (*Columba livia*) and zebra finch (*Taeniopygia guttata*) to assess the origin of the potential of the involved structures of the visual pathways (19–21). The feasibility of FVEP recording has also been evaluated in four species of diurnal birds of prey: Harris’s hawk (*Parabuteo unicinctus*), lanner falcon (*Falco biarmicus*), gyrfalcons (*Falco rusticolus*), and saker falcon (*Falco cherrug*) under general anesthesia (21). Despite the small number of birds involved in the study, FVEPs were considered safe, easy to perform and useful for the assessment of visual function. The case reported herein provides the first description of a traumatic brain injury evaluated using BAER testing as an indirect test to assess the brainstem as well as the hearing integrity in a pet tawny owl. Additional examinations, such as flash visual evoked potential (FVEP) recording and CT scan, were also performed.

## 2 Case description

**Figure 1:**
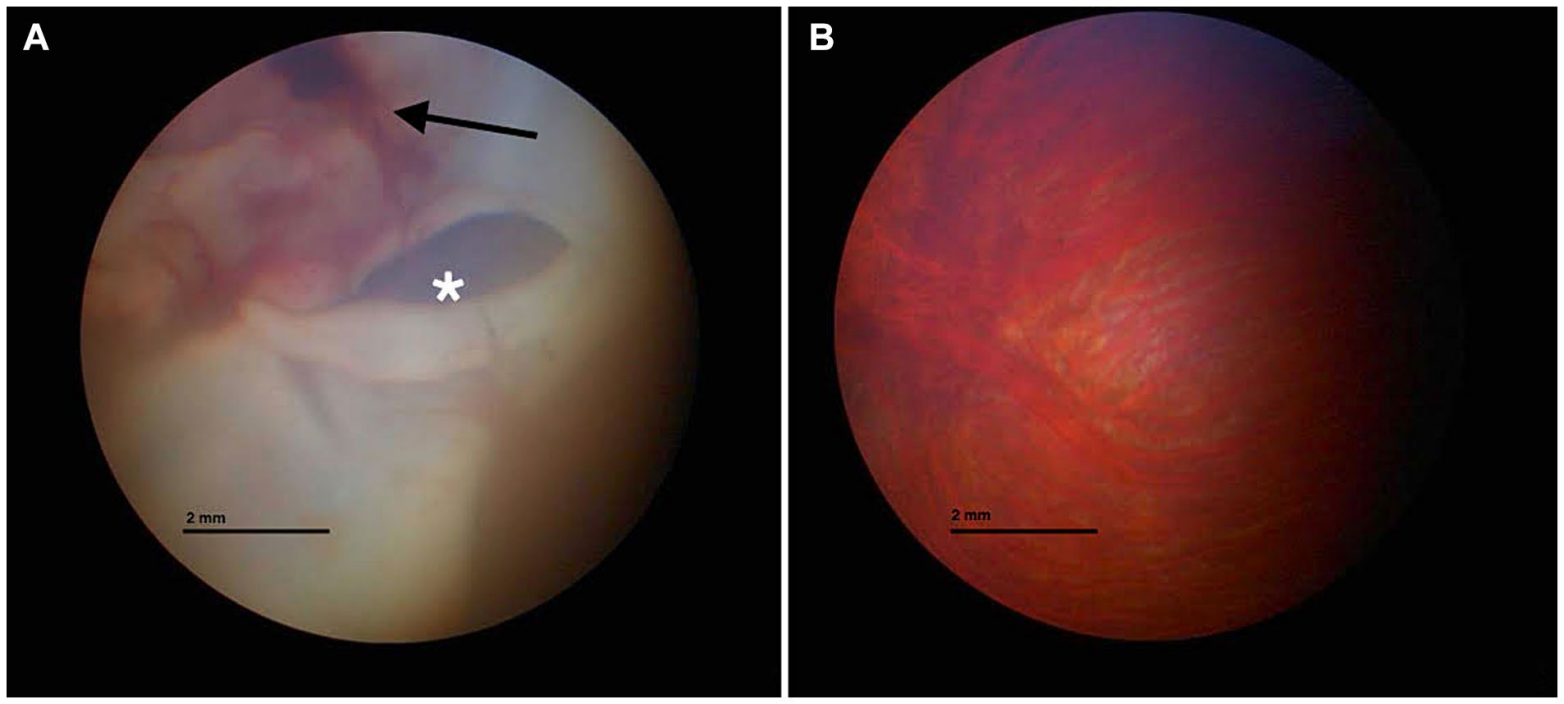
Fundus inspection of the left **(A)** and right eyes **(B)**. **(A)** Vitreal hemorrhage (black arrow) and retinal detachment (white asterisk) are visible. **(B)** Normal fundus appearance.

A captive-bred, adult male pet tawny owl (*Strix aluco*) weighing 500 g was presented to a veterinary hospital in Parma because of a history of head trauma against a window while attempting to escape out of the house. The trauma occurred almost 13 h before the clinical examination. Upon clinical examination, the animal appeared to have good nutritional status but was unable to fly and exhibited obtunded senses. A large, dark red hematoma extending into the acoustic meatus was noted in the left ear. At the opening of the oral cavity, the palatine fissure exhibited small blood clots. For a duration of 12 h, the owl was hospitalized and placed under oxygen in an incubator at 30°C (100% sat, 4 L/min), and warm fluids (Ringer solution) S.a.l.f. Spa, Via Guglielmo Marconi, 2, 24,069 Cenate sotto BG (Italy) (50 mL/kg, SC) (5) and vitamin B12 (Dobetin 500 μg/mL injectable Via Amelia 70, 00181, Roma, Italia) (0.5 mg/kg IM) (19) were administered subcutaneously. A single dose of tramadol [Nargesic^®^, Acme Srl, Via Portella della Ginestra 9, 42,025 Z.I. Corte Tegge (RE), Italy] was administered at 15 mg/kg PO. Meloxicam at 0.5 mg/kg (Metacam^®^ 5 mg/mL, Boehringer Ingelheim S.p. A, Noventana, Italy) was administered orally twice a day. Gavage feeding was performed [Emeraid Exotic Carnivore, 30 mL orally, every 6–8 h, Slow Global, Corso Liberta’ 262, 13,100 Vercelli (VC), Italy] for an additional 12 h. Once stabilized, the tawny owl was placed under gaseous general anesthesia (2% isoflurane) (Isoflo^®^ Zoetis Italia S.r.l, Via Andrea Doria, 41 M, 00,192 Roma RM), and two radiograph projections (latero-lateral and dorsal-ventral) were performed. No fractures or other pathological findings were noted. A 2 mL sample of venous blood was drawn from the ulnar vein for a complete blood cell (CBC) count and biochemical analysis, demonstrating no alterations compared with the published reference values for the species (22). An ophthalmic exam was performed. The right eye examination was unremarkable. The left pupil was mydriatic, and no sign of vision was observed. Indirect ophthalmoscopy revealed diffuse retinal edema and detachment. The left pecten was hypotrophic compared with the right pecten. Fundus inspection revealed vitreal hemorrhages and retinal detachments (Figure 1). For two additional days, the owl continued to receive supportive care with subcutaneous fluid therapy of 20 mL NaCl 0.9% followed by intravenous infusion of Ringer’s lactate and 5-mL tube feeding with EmerAid Carnivore (EmerAid Intensive Care Carnivore, Cornell, IL, United States) q12h. Meloxicam and tramadol were used to minimize pain and inflammation (Topalgic 100 mg/mL, Sanofi Aventis, Paris, France) at 30 mg/kg PO q12h.

**Figure 2:**
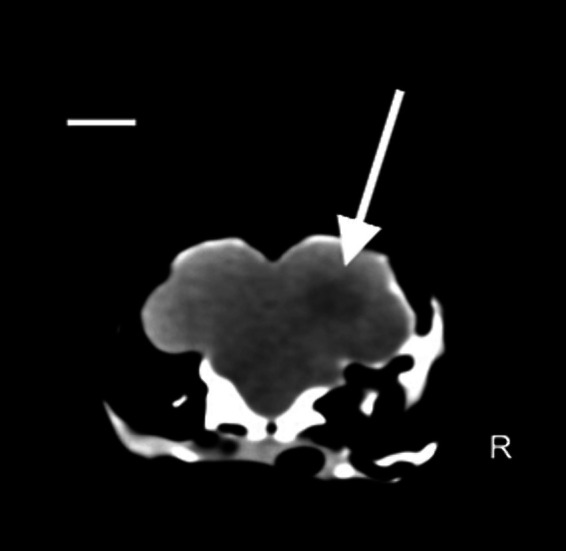
Coronal plane image with soft tissue algorithm in the brain window. Scale bar = 8 mm. A hypoattenuating ovalar intra-axial lesion with ill-defined margin is observed at level of parieto-temporal area at the level of the right cerebral hemisphere (white arrow) (Pitch 0.562, slice thickness: 1.25 mm).

The clinical state of the tawny owl remained stable. However, to advance the diagnostic process and gain crucial insights into vision prognosis, a comprehensive whole-body CT scan was selected. The owl was placed under gaseous general anesthesia (2% isoflurane), and a total body CT scan without the use of contrast media was performed using a single-slice spiral scanner (SOMATOM EMOTION, Siemens Spa, Milano, Italy). An intraxial, ill defined, ovalar and hypoattenuating (75 HU) lesion was reported in the right cerebral hemisphere. No skull fractures were identified. Based on these findings, post-traumatic cerebral edema was hypothesized, such as from severe non-hemorrhagic cerebral contusion (Figure 2).

**Figure 3:**
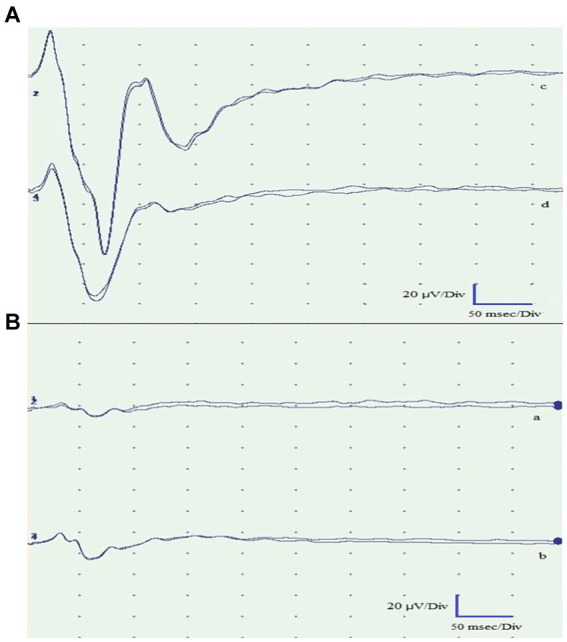
Flash visual evoked potentials of the right eye **(A)** and left eye **(B)**. Traces a and c were obtained by light stimulation with the eyes open. Traces b and d were obtained with the eyes closed. The recorded traces from the left eye exhibited pathological findings, demonstrating the absence of peaks in both stimulations. Conversely, the traces obtained from the right eye were normal and exhibited the expected peaks.

To better investigate this result, FVEP tests were performed. The test was recorded using Electromyography and Evoked Potentials Systems (MyoHandy, Micromed, Treviso, Italy). The animals were placed in sternal recumbency with their heads elevated using a support to facilitate proper light stimulation. VEPs were recorded using a bipolar method utilizing stainless-steel needle electrodes (size 10 × 0.25 mm). These electrodes were applied subcutaneously at specific locations: the midline of the forehead between the eyes (Fpz) served as the negative electrode, the occipital region on the nuchal crest (Oz) served as the positive electrode, and a ground electrode was positioned subcutaneously at Cz (vertex). Before the recordings, mydriatic drugs were not administered, as adequate mydriasis was achieved as part of the anesthetic plan. Throughout the recording sessions, artificial tears (Epigel^®^, Trebifarma s.r.l, Viale Ammiraglio Giorgio Des Geneys, 60R, 16,148 Genova GE) were applied to keep the eyes moistened. The recordings took place in a quiet and well-lit room with the animal exposed to light for at least 30 min before the session to ensure proper adaptation of the eyes to the light conditions. Each stimulus was a flash of light at an intensity of 10,000 millicandelas per square meter (mcd s/m*^2^*). These flashes were generated using a xenon lamp photostimulator (Flash Stimulator, Micromed^®^ Treviso, Italy). This device produces bright white light that closely resembles natural sunlight. To trigger the xenon lamp, the Flash Stimulator was directly connected to MyoHandy^®^ (Micromed group, Via Giotto, 2, 31,021 Zona Industriale S.p.z. TV, Italy) device. The xenon lamp unit was positioned 15 cm in front of the eye being examined. The eyelid of the eye under examination was gently opened, and the contralateral eye was covered with a black eye patch to ensure that the light stimulus was specific to the eye under observation. Each eye underwent two consecutive series of stimulations. The first series involved flashes at a frequency of 1 Hz, followed by the second series with flashes at a frequency of 6 Hz. There was a 2-min interval between the two series to allow for adequate recovery and adaptation between stimulations. The duration of the light stimulus was approximately 20 μs. The traces of the left eye revealed no peaks in either series of stimulations, reflecting an altered functional integration within the visual pathway (Figure 3), whereas traces of the right eye were normal.

The subject underwent the BAER examination, performed with acoustic stimulation in the form of Click Standard, with an intensity of 80 dB nHL. This test was performed under the same anesthesia, as all the monitored parameters were stable. Subcutaneous monopolar scalp electrodes were positioned based on previously described conditions in healthy Strigiformes birds (23): a positive electrode on the vertex, a negative electrode near the auditory meatus and a neutral electrode in the retro-occipital area. A monaural click stimulus at 80 dB, able to generate the square wave, was sent to the patient, and only the rise/fall time parameter was considered, usually very fast: slew rate measured in μV/s. The duration of the stimulus was approximately 10 ms, each recording was obtained from an average of approximately 500 stimuli, and all tracings were recorded in duplicate to ensure repeatability of the graph and to eliminate any artifacts.

**Figure 4:**
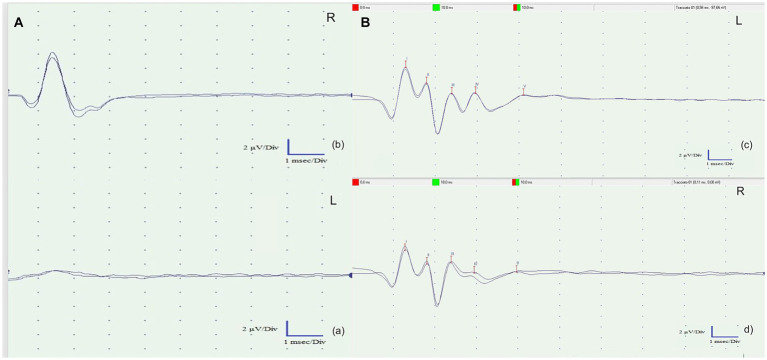
**(A)** Brainstem auditory evoked response (BAER) tests of the left ear **(A)** and the right ear (b). Both recorded tracings exhibited a complete absence of waves III to V, suggesting a likely overall involvement of the brainstem structures, as indicated by the clinical evaluation. However, on the left side (a), there is probable involvement of the inner ear. This hearing impairment resulted in the disappearance of wave I (cranial nerve VIII) and subsequent waves in the recordings. **(B)** Left (c) and right (d) BAER tracks of a clinically healthy tawny owl (*Strix aluco*) used to compare the BAER tracks obtained from the patient. The presence of five physiological waves (I–V) is evident.

As observed in Figure 4, in comparison with a physiological tracing from a healthy, captive-bred tawny owl obtained from another owner (Figure 4B), both tracings obtained were characterized by the complete absence of waves from III to V as a suspected general involvement of the brainstem structures, according to the clinical evaluation. However, on the left, as suspected given the involvement of the internal auricular portions, there is hypoacusis at 80 dB, resulting in the disappearance of wave I (cranial nerve VIII) and subsequently the disappearance of the others.

The length of the entire diagnostic procedure was approximately 20 min. The tawny owl died during recovery from anesthesia without any evident cause. Necropsy was refused by the owner.

## 3 Discussion

Head trauma in birds is one of the most common causes of neurological disfunction (1, 24, 25) and is often related to collisions (24–28). Comprehensive and meticulous observations, along with several controlled experiments, have demonstrated that, as a general trend, birds do not perceive clear or reflective glass or plastic panes as barriers to be avoided (24–28). Hence, it is unsurprising that the same holds true for pet birds kept in domestic environments. Fractures are a common finding in wild birds that have experienced blunt trauma (24–28). In the case described herein, skull fractures were not evident on either radiographic or CT scan examination. As the traumatic event occurred indoors, it is possible that the short distance covered by the tawny owl prior to hitting the glass was not sufficient to cause bone injuries. However, as literature regarding indoor blunt traumas in birds is scarce, every hypothesis about this finding remains speculative. Ocular injuries are a prevalent aspect of head trauma in birds, with an incidence of over 30% among traumatized avian individuals (29, 30), because of the large orbit, which accommodates their relatively large eyes and scleral ossicles (30). Owl species tend to experience ocular lesions more frequently compared with other birds of prey, primarily due to the unique anatomical and orbital structure of their eyes (31). In this case, we observed vitreal hemorrhages, retinal detachments and hypotrophy of the left pecten relative to the right pecten during the fundus inspection. CT scan revealed a blurry hypoattenuating area within the right cerebral hemisphere. A significant region of cerebral edema was present throughout much of the deep interior of the right cerebral hemisphere. Although the cause is not definitively known, a non-hemorrhagic cerebral contusion is a possible etiology. Bilaterally, there was no visualization of ventricles, sulci, fissures, or cisterns, suggesting increased intracranial pressure. Therefore, differential diagnosis should include cerebral edema secondary to cerebral contusion due to the blunt trauma (32). CT scans have proven to be clinically useful for imaging the brain in birds (33). The decision to perform CT instead of MRI was made for two reasons. The first is related to the duration of anesthesia necessary for the different procedures, with CT needing only a short duration because the procedure is so fast, relative to MRI, which is lengthy As reported in dogs, prolonged anesthesia times after head trauma promote an increased risk of anesthesia and post anesthesia complications (8, 34). Increased intracranial pressure from the administration of anesthetic drugs would, based on the Monroe Kelly doctrine, result in an increased risk of cerebral or cerebellar herniation (35). The second aspect is related to the better evaluation of acute hemorrhage and bone fracture with CT relative to MR imaging, as reported in dogs and cats (36). The limitation of this procedure was related to the absence of contrast medium injection that restrict evaluation of other pathologic areas, if present, or a well clarification of which kind of lesion was observed. Among disorders affecting the posterior eye segment (fundus oculi) in birds, trauma-related hemorrhages are the most frequently diagnosed (37). When the choroid and retina experience traumatic injury, there is a rapid exudation of fluid and cells into the subretinal space, ultimately causing retinal detachment (38). Moreover, the energy transfer from the rigid sclera to the internal structures of the eye could have had a profound impact on the integrity of the retina. In fact, as energy propagates through the semiliquid retinal tissues, it exerts a disruptive force on the most delicate layer of the retina (39). A characteristic of the avian retina that contributes to a higher risk of damage in trauma cases is its avascular nature. When subjected to impact, the globe expands along the equatorial plane, causing destabilization of the uveoscleral interface. Unlike in vascularized retinas, the avascular structure lacks the tensile support provided by the retinal vascular network. This absence of support makes the otherwise semiliquid retinal tissue susceptible to additional shearing, especially in response to oscillating, lower-amplitude pressure wave reflections that occur following the initial short-duration, high-amplitude shock waves typical of blunt traumas (40).

Flash visual evoked potential measurements performed on the left eye exhibited no peaks in either series of stimulations, indicating an altered functional integration within the visual pathway. In birds, invasive techniques involving visual evoked potentials were employed to discern the origin of the potential from individual structures within the visual pathways (17, 18). In dogs, the utility of the fVEP test lies in its ability to correlate the function of specific structures in the visual pathway with the presence of distinct peaks on the recordings. This allows for the identification of the neuroanatomical location of visual lesions, either by the absence of certain potentials or by observing an increase in their latency times (41). Drawing similar conclusions in birds of prey is challenging due to the lack of precise data on their neurofunctional anatomy (20). Despite the existence of significant neuroanatomical similarities with the visual pathways of mammals, which could enable the formation of parallel hypotheses, the absence of accurate information hinders such definitive assessments. Indeed, in birds of prey, there are two parallel visual pathways known as the tectofugal and thalamofugal pathways. The tectofugal pathway corresponds to the extrageniculostriate system observed in mammals, especially in primates, whereas the thalamofugal pathway corresponds to the geniculostriate system. In detail, the tectofugal (collothalamic) pathway consists of optic nerve axons that intersect to varying degrees depending on the species to form the optic chiasm. These fibers then travel to the optic tectum, followed by the round nucleus of the thalamus and finally to the extrastriate cortex (41). With respect to physiological BAER values in birds, literature is lacking. Based on a recent study by Garello et al. (23) on 28 clinically healthy nocturnal raptors (including 4 tawny owls), the most prominent and consistent waves on tracks were the P1, the P2, and the P3. These peaks corresponded to the stimulation of the eighth cranial nerve, the cochlear nucleus, and the lower olivary complex, respectively. These findings were consistent with the track obtained from a clinically healthy tawny owl used as a comparison. The patient exhibited a complete absence of waves III to V, suggesting potential overall involvement of the brainstem structures based on the clinical evaluation. Notably, on the left side, there was probable involvement of the internal auricular portions, leading to 80 dB hearing loss, which resulted in the disappearance of wave I (cranial nerve VIII) and subsequently all other waves. The death of the tawny owl during the recovery from anesthesia could be related to the severity of the injuries, to the anesthesia itself, or both. In fact, due to its impact on central and peripheral control mechanisms, isoflurane has the potential to induce cardiac and respiratory depression in both mammals and birds (42). Moreover, isoflurane has been shown to have dose-dependent respiratory depressant effects in several avian species (43–45). It is possible that the length of the entire diagnostic procedure (approximately 20 min) might have affected the already weakened nervous system. Furthermore, the newly developed Avian Modified Glasgow Coma Scale, which is useful as a prognostic indicator in cases of raptors presenting with head trauma, was not available at the time of the described case (6). The authors cannot state with certainty whether or not this new scale would have provided a positive prognostic index for the case described so far. However, this demonstrates the existence of an additional tool for defining the diagnostic and therapeutic plan in the course of head trauma in avian species.

## 4 Conclusion

Head trauma can have a profound impact on birds, leading to notable neurological impairments that affect their behavior, mobility, and cognitive abilities. Utilizing diagnostic tools such as FVEP recordings, BAER tests, and CT scans can be valuable in a clinical setting to assess and understand the extent of neurological damage. It is crucial for clinicians to comprehend the causes and neurological manifestations of avian trauma to ensure effective prevention, accurate diagnosis, and appropriate treatment for affected birds.

## Data Availability

The raw data supporting the conclusions of this article will be made available by the authors, without undue reservation.
